# ZBTB Transcription Factors: Key Regulators of the Development, Differentiation and Effector Function of T Cells

**DOI:** 10.3389/fimmu.2021.713294

**Published:** 2021-07-19

**Authors:** Zhong-Yan Cheng, Ting-Ting He, Xiao-Ming Gao, Ying Zhao, Jun Wang

**Affiliations:** ^1^ Institutes of Biology and Medical Sciences, Soochow University, Suzhou, China; ^2^ Department of Pathophysiology, School of Biology and Basic Medical Sciences, Soochow University, Suzhou, China

**Keywords:** thymic T-cell development, T-cell differentiation, T-cell effector function, ZBTB proteins, transcriptional regulation

## Abstract

The development and differentiation of T cells represents a long and highly coordinated, yet flexible at some points, pathway, along which the sequential and dynamic expressions of different transcriptional factors play prominent roles at multiple steps. The large ZBTB family comprises a diverse group of transcriptional factors, and many of them have emerged as critical factors that regulate the lineage commitment, differentiation and effector function of hematopoietic-derived cells as well as a variety of other developmental events. Within the T-cell lineage, several ZBTB proteins, including ZBTB1, ZBTB17, ZBTB7B (THPOK) and BCL6 (ZBTB27), mainly regulate the development and/or differentiation of conventional CD4/CD8 αβ^+^ T cells, whereas ZBTB16 (PLZF) is essential for the development and function of innate-like unconventional *γ*δ^+^ T & invariant NKT cells. Given the critical role of T cells in host defenses against infections/tumors and in the pathogenesis of many inflammatory disorders, we herein summarize the roles of fourteen ZBTB family members in the development, differentiation and effector function of both conventional and unconventional T cells as well as the underlying molecular mechanisms.

## Introduction

### Early T-Cell Development in the Thymus

Most immune cells are generated during hematopoiesis through a stepwise branching where multipotent hematopoietic stem cells (HSCs) divide and differentiate into different progenitors with a restricted set of fates at later stages, such as lymphoid-primed multipotent precursors/early lymphoid precursors (LMPPs/ELPs) and common lymphoid progenitors (CLPs) (1). Upon entering the thymus, these thymus-settling precursors, presumably derived from LMPPs/ELPs or CLPs, proliferate extensively and initiate the T cell development program driven by environmental signals, like SCF (stem cell factor), NOTCH & IL-7 signalings, and a panel of transcription factors (TFs), including TCF7 (transcription factor 7), GATA3 (GATA binding protein 3), BCL11B (B cell leukemia/lymphoma 11B), and TCF3 (E2A), that act sequentially/collaboratively at multiple steps (2, 3).

**Figure 1:**
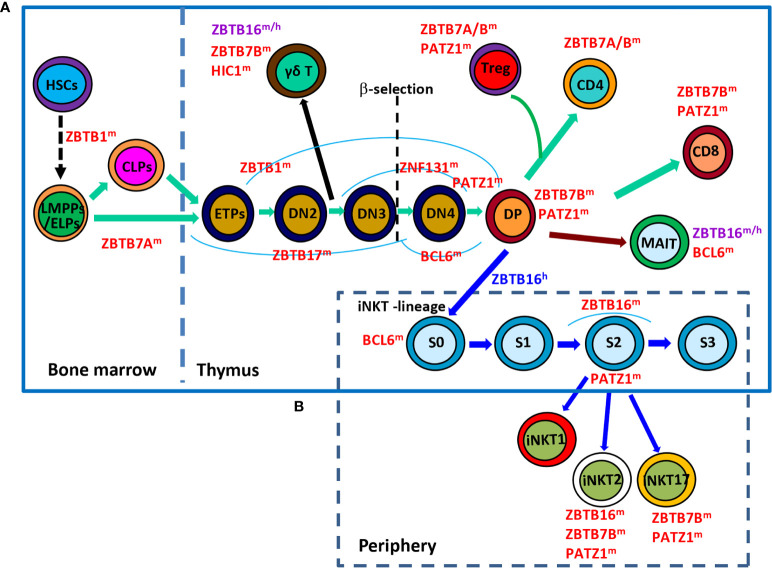
Roles of ZBTB proteins in early T-cell development. **(A),** a schematic view of the stages most affected by ZBTB proteins along the early T-cell development program. ZBTB7A prevents the pre-mature differentiation of developing HSCs into DP T cells in the BM, while ZBTB1, ZBTB17, ZNF131, BCL6 and PATZ1 regulate the early development of conventional αβ^+^ T cells before the DP stage. Moreover, ZBTB16, ZBTB7B and HIC1 are important for the development/function of *γ*δ^+^ T cells which diverge from the conventional T-lineage program at the DN2 stage. Since the DP stage, PATZ1, ZBTB7B & ZBTB7A modulate the differentiation of conventional CD4/CD8 SP cells and Treg cells, whereas ZBTB16 and BCL6 promote the development of unconventional MAIT cell and iNKT cells from DP cells. **(B)**, a simplified overview of the development of iNKT cells in thymus as well as their functional maturation in the periphery. BCL6 promotes the transition of stage 0 (S0) iNKT cells into stage 1 (S1), after which ZBTB16 promotes their intrathymic expansion and effector differentiation. Moreover, PATZ1 and ZBTB7B fine-tune the subset differentiation of iNKT cells. ZBTB16 regulates iNKT cell development in humans as well. ZBTB proteins with the superscript ‘m’, ‘h’ or ‘m/h’ in the upper right corner indicate that these proteins are ascribed to mice, humans or both, respectively. S, stage.

Within the thymus, thymocytes sequentially transit through CD4^-^CD8^-^ double-negative (DN) DN1 (CD44^+^CD25^-^), DN2 (CD44^+^CD25^+^), DN3 (CD44^-^CD25^+^), DN4 (CD44^-^CD25^-^), and CD4^+^CD8^+^ double-positive (DP) stages on the way to becoming mature CD4^+^ or CD8^+^ single-positive (SP) αβ^+^ T cells (**Figure 1A**). The interaction of NOTCH1 molecule on thymocytes with its ligand delta-like (DLL) 4 on thymic epithelial/stromal cells promotes the survival and developmental progression of early T cell progenitors (ETPs, KIT^high^CD44^+^CD25^-^), a tiny subset of DN1 cells expressing high level of KIT (receptor for SCF), into the subsequent DN2 and DN3 stage until β-selection mainly *via* upregulating HES1, TCF7 and GATA3, TFs essential for specification of the T cell lineage (3). HES1 and GATA3 remove the non-T cell fate potentials of ETPs and early DN2 cells (4, 5), while TCF7 not only directly activates a subset of T cell signature genes (*Gata3*, *Bcl11b*, *Il2ra*, and *Rag2*, etc*)*, but also appears to function as a pioneer-like factor to establish a T-cell specific chromatin landscape in developing thymocytes (6, 7). Moreover, E proteins, in particular TCF3 (E2A), control and collaborate with NOTCH1 to activate many other indispensable T cell lineage genes, such as *Rag1/2*, *Ptcra* (encoding pre-TCRα/pTa), *Cd3g/d/e* (encoding CD3*γ*/δ/ε chains, respectively) and *Tcrb*, during early T cell development (8). BCL11B is rapidly upregulated in DN2 thymocytes, partially by TCF7 & GATA3, and promotes their downstream differentiation through upregulating essential components of pre-TCR in DN2 cells, assisting the recombination and expression of TCR-β in DN3 cells, and promoting the survival of DP cells as well as the positive selection of CD4 or CD8 SP mature αβ^+^ T cells (9). Upon recognizing self-antigens presented by thymic dendritic cells or medullary epithelial cells with intermediate affinities, a minor subset (~ 1%) of developing CD4^+^ SP thymocytes acquires Foxp3 expression and becomes regulatory T (Treg) cells (10).

Although originated from a common thymic progenitor, the *γ*δ^+^ T lineage starts to diverge from αβ^+^ T cells at the DN2 stage. It is proposed that DN cells receiving weak TCR signals serve as a precursor pool for conventional αβ^+^ T cells, while those perceiving strong TCR signals are committed to the *γ*δ^+^ T lineage and become functionally mature without progression through the DP stage (**Figure 1A**) (11, 12). Strong TCR signaling-induced downstream events, such as high levels of ERK (extracellular signal-regulated kinase), EGR (early growth response) and ID3 (inhibitor of DNA binding 3), favor the development of *γ*δ^+^ T cells rather than their αβ^+^ counterparts (12).

In addition, developing thymocytes diverge at the DP stage to give rise to two additional unique subsets of T lymphocytes, invariant nature killer T (iNKT) cells and mucosal associated invariant T (MAIT) cells (**Figures 1A, B**). iNKT cells are characterized by their invariant TCRs comprised of a single invariant α chain (Vα14-Jα18 in mice, and Vα24-Jα18 in humans) in combination with certain β chains (Vβ8, Vβ7 or Vβ2 in mice, and Vβ11 in humans) that specifically recognize lipid antigens in the context of CD1d (13, 14). Notably, iNKT cells are positively selected by CD1d-expressing DP cells themselves, instead of epithelial cells that drive the selection of conventional αβ^+^ T cells. Moreover, the cooperative engagement of the homophilic receptors of the SLAM (signaling lymphocytic-activation molecule) family member SLAMF1 & SLAMF6, which leads to the downstream recruitment of the adaptor SAP (SLAM-associated protein) and the SRC kinase FYN, is indispensable for the characteristic expansion and differentiation of iNKT cells post positive selection (13). Likewise, MAIT cells express semi-invariant αβ TCRs with a canonical Vα19-Jα33 paired with Vβ8/Vβ6 in mice or Vα7.2-Jα33 paired with Vβ2/Vβ13 in humans that engage MHC-I related protein 1 (MR1), accumulate in the intestinal lamina propria and are capable of instantly releasing IL-4, IFN-*γ* and/or IL-17 upon activation (14, 15). Akin to iNKT cells, MAIT cells are positively selected by MR1-expressing DP thymocytes with the reliance of SLAM interactions as well (14).

### Differentiation and Function of T Cells in the Periphery

**Figure 2:**
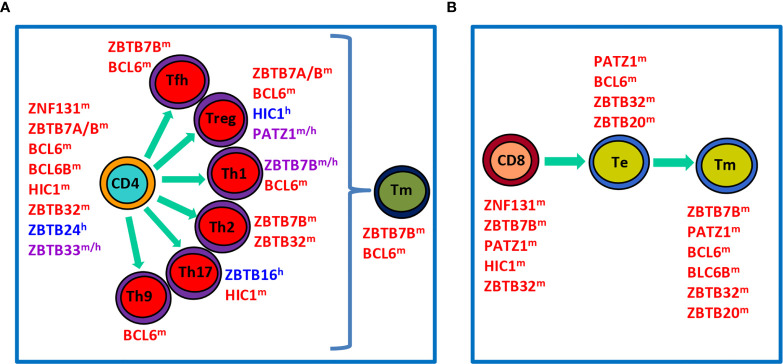
Roles of ZBTB proteins in the proliferation, differentiation and function of conventional T cells in the periphery. **(A),** a schematic view showing that ZBTB proteins regulate the survival, proliferation and/or function of undifferentiated CD4^+^ T cells, differentiated effector Th subsets, or Tm cells. **(B)**, a schematic view illustrating roles of ZBTB proteins in CD8^+^ T cells along their differentiation into Te and final Tm process. ZBTB proteins with the superscript ‘m’, ‘h’ or ‘m/h’ in the upper right corner indicate that these proteins are ascribed to mice, humans or both, respectively.

After maturation in the thymus, T cells migrate into the periphery to exert their effector functions. Upon encountering the cognate antigen, naive CD4^+^ T cells proliferate and differentiate into various Th (T helper) subsets characterized by expressions of distinct TFs and signature cytokines (**Figure 2A**). This process is dictated by the strength of TCR signal and the presence of polarizing cytokines in the microenvironment during the priming of naive T (Tn) cells. For instance, strong TCR signaling plus IL-12/IFN-*γ* predisposes the generation of TBX21 (Tbet)^+^ Th1 cells, whereas low-dose peptide with IL-4/IL-2 favors the differentiation of GATA3^+^ Th2 cells (16). Likewise, CD8^+^ T cells are capable of differentiating into different T cytotoxic (Tc) subsets under similar cytokine conditions as well (17). By producing unique sets of cytokines, different subsets of Th & Tc cells play critical roles in orchestrating host defences against infections & tumors, as well as immune responses underlying various inflammatory disorders (16, 17). After the elimination of antigens, the effector immune responses contract and only a minority of effector T (Te) cells will differentiate into long-lived memory T (Tm) cells capable of mounting a more rapid and robust response upon antigen reexposure (**Figures 2A, B** **)**. In addition to cytokines and TFs, recent studies highlight that cell metabolism, such as glycolysis and fatty acid oxidation, represents another important regulator of the survival, activation, proliferation, differentiation and effector function of CD4^+^ & CD8^+^ T cells (18, 19).

Unlike conventional αβ^+^ T cells bearing a diverse TCR repertoire and residing predominantly in secondary lymphoid organs in the periphery, *γ*δ^+^ T, iNKT and MAIT cells express a highly restricted TCR and localize mainly in the liver, epithelial layers of the skin, reproductive tract or gut mucosa. Upon recognizing antigens, for instance, glycolipids by iNKT and microbial metabolites by MAIT cells, these unconventional T cells rapidly produce copious amounts of cytokines that are normally only made by fully differentiated conventional Te cells (13, 20). Moreover, some iNKT cells are able to secret IL-4 at steady state, and coproduce both IFN-*γ* and IL-4 upon activation (13). As such, these unconventional T cells, with both innate- and adaptive-like characteristics, bridge innate and adaptive immunity.

### ZBTB Proteins

**Figure 3:**
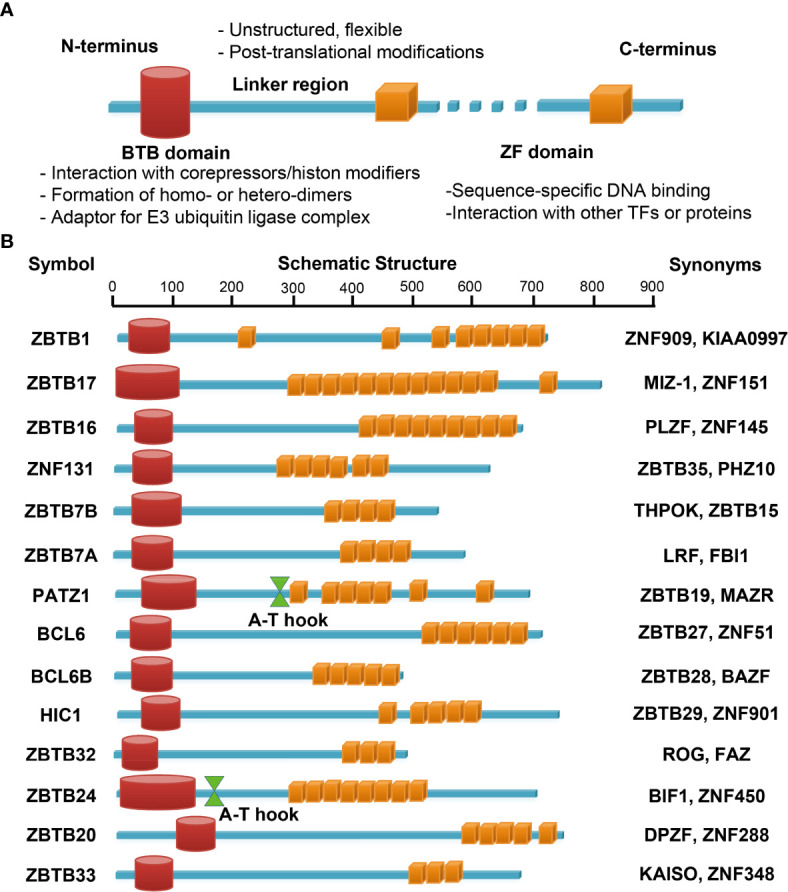
Schematic illustrations of the structure and main function of ZBTB protein domains. The N-terminal BTB domain mainly mediates the protein-protein interactions, while the C-terminal ZFs mainly mediate the bindings to DNA. The ZF domains may interact with other TFs or proteins as well. The linker domain is unstructured, often flexible and targeted for posttranslational modifications **(A)**. **(B)** functional domains of the fourteen ZBTB proteins discussed in this review. PATZ1 and ZBTB24 contain an additional A-T hook domain with DNA-binding ability.

ZBTB (Zinc finger and BTB domain containing) proteins are an evolutionary conserved family of transcriptional regulators with about 60 different members. ZBTB proteins are characterized as having several C-terminal C_2_H_2_/Krüppel-type zinc finger (ZF) domains and an N-terminal BTB (broad-complex, tram-track and bric-a-brac) domain (21, 22) (**Figure 3A**). The BTB domain binds to different corepressors and histone modification enzymes, including BCoR (BCL6 corepressor) or NCoR-1/2 (nuclear receptor corepressor-1/2) complex, NuRD (nucleosome remodeling deacetylase) and HDAC (histone deacetylase) etc, and thus remodels the chromatin structure and accessibility. This domain also mediates the formation of homo- or hetero-dimers among ZBTB proteins, and may serve as an adaptor for cullin 3-based E3 ubiquitin ligase complex, both of which are critical for their stability, localization and transcriptional activity inside cells (21, 22). By contrast, the ZF domains directly bind to specific sequences in the genome and hence determine the transcriptional specificity of ZBTB proteins, albeit they may interact with other proteins as well (21, 23). For instance, by association with PARP1 *via* its ZF domains, ZBTB24 protects PARP1 from degradation at DNA breaks, and thereby promotes the error-free nonhomologous end-joining (NHEJ) process independent of its transcriptional activity (23). Notably, ZBTB proteins may regulate gene transcription in a cell type/subset specific fashion, as genome-wide analysis has revealed that the binding motifs of BCL6 (ZBTB27) differ substantially in primary Tfh (T follicular helper), Th9, macrophages and B cells (24). The poorly conserved and flexible linker region, lying between the BTB and ZF domains, is often targeted for posttranslational modifications, and thereby modulates the stability and flexibility of ZBTB-protein/DNA complex (22). Finally, some ZBTB proteins contain an additional A-T hook domain, a DNA-binding motif capable of interacting with the minor groove of AT-rich sequences (**Figure 3B**) (25).

Numerous studies have shown that ZBTB proteins are implicated in diverse cellular processes, such as DNA damage responses and cell-cycle progressions, and a variety of developmental events, including gastrulation, organ formation and HSCs fate determination (21, 26). So far at least 13 *Zbtb* genes have been demonstrated to have critical roles in HSCs lineage determination and differentiation, among which seven ZBTB members regulate B-cell development & function (21, 22). Studies have highlighted a central and indispensable role of ZBTB proteins in nearly all aspects of T cell biology. Here we will summarize and discuss recent findings on 14 ZBTB proteins, as illustrated in **Figure 3B**, with reported roles in the development, differentiation, and effector function of conventional and/or innate-like unconventional T cells (**Figures 1**, **2**).

## ZBTB Proteins Mainly Involved in Thymic T-Cell Development

### ZBTB1

Although ZBTB1 is dispensable for the generation of immune progenitors from HSCs, its deficiency results in a severely impaired generation of mature lymphoid, and to a less extent, myeloid cells in mice (27–29). ZBTB1 knockout mice are nearly absence of T cells as their precursors fail to progress beyond the DN1 stage in thymus, and the very few splenic T cells exhibit a complete lack of proliferative and cytotoxic function (27). ZBTB1 maintains the genome integrity in replicating cells by inducing the phosphorylation of CHEK1, which blocks DNA synthesis for lesion repair, thereby preventing TP53-mediated apoptosis (30). Moreover, ZBTB1 associates with the TRIM28/NuRD complex and guides it to DNA damage sites, resulting in chromatin remodeling and initiation of translesion DNA synthesis (31). However, prevention of apoptosis by BCL2 overexpression or TP53 deficiency only partially rescues the early stages of T-cell development till the DN3 or DP stage, respectively, in ZBTB1-deficient mice (**Figure 1A**) (30). Given that ZBTB1 directly represses cAMP signaling, the activation of which abrogates TCRα gene rearrangements, and that TCRα-null thymocytes do not proceed beyond the DP stage (32, 33), promoting TCRα rearrangements *via* inhibiting cAMP signaling may underlie the apoptosis-independent function of ZBTB1 at later stages of T-cell development in the thymus. Recently, a reciprocal suppression between ZBTB1 expression and IL-7Rα signaling has been discovered (34), but the detailed underlying molecular mechanisms and the impact of this regulation on developing thymocytes remain to be determined. Additionally, ZBTB1-deficient mice lack ROR*γ*t^+^NKp46^+^ innate lymphoid cells (ILCs) in the intestine, which can not be reversed by BCL2 overexpression and TP53 deficiency (29). Of note, despite the similar extent of T-cell deficiency, ZBTB1-knockout mice have increased numbers of short-term HSCs, multi-potent progenitors and CLPs (27), while scanT mice with a single substitution of the conserved cysteine (C47R) in the BTB domain of ZBTB1 do not (28). This indicates that ZBTB1 protein in scanT mice still retain some residual function sufficient to maintain HSCs numbers and differentiation.

### ZBTB17 (MIZ-1)

ZBTB17, initially identified as MYC-interacting ZF protein 1 (MIZ-1), is indispensable for early embryonic development during gastrulation (35). Mice with a transcriptionally inactive ZBTB17 (Vav-Cre mediated deletion of the BTB domain) in hematopoietic cells display a severely impaired lymphoid, but not myeloid, development (36, 37). Although ZBTB17 does not regulate genes specifying the T-cell lineage in LMPPs/ELPs, its deficiency results in an almost entirely lack of ETPs as well as a severe block of DN to DP transition during thymic pre-T cell development (37, 38). ZBTB17-deficient DN pro-T cells are prone to apoptosis as they fail to relay signals from IL-7R and thus upregulate the anti-apoptotic gene BCL2. ZBTB17 represses the expression of the STAT5 inhibitor SOCS1 (suppressor of cytokine signaling-1) by directly binding to its initiator site, thereby promoting IL-7-induced STAT5 phosphorylation and BCL2 upregulation in DN cells. However, inhibition of SOCS1 or overexpression of BCL2 only restores the differentiation of ZBTB17-deficient pro-T cells till the DN3 stage, indicating that ZBTB17 regulates the DN3-to-DN4 pre-T cell transition *via* different mechanisms (37).

Despite the normal expression of genes required for the generation of pre-TCR and V(D)J recombination such as *Rag1/2*, *Ptcra* and *Dntt* (encoding terminal deoxynucleotidyl transferase/Tdt), few DN3/DN4 cells with surface pre-TCR are present in mice with unfunctional ZBTB17, partially because of TP53-induced apoptosis and cell cycle arrest (39, 40). ZBTB17 indirectly inhibits the translation of TP53 by activating ribosomal protein L22, which binds to the mRNA of TP53 and represses its translation (40). Alternatively, ZBTB17 is also capable of directly interacting with TP53 *via* its ZF domains, and thereby diminishes TP53’s binding to target promoters (41). As the coexpression of both rearranged TCRαβ and BCL2 or ablation of TP53 rescues the ZBTB17-deficient DN3/4 cell numbers and their further differentiation into DP cells, ZBTB17 seems to be required to ensure proper TCRβ expression and TP53 activity at the pre-TCR “β-selection” checkpoint (**Figure 1A**) (39, 40). Notably, the normal thymic T-cell development in mice when ZBTB17-inactivation is only accomplished at the DN4 stage (mediated by Lck-Cre) indicates that ZBTB17 has little effect at later thymocyte differentiation/maturation beyond the DN4 stage (37, 39). Alternatively, other factors may compensate for the loss of ZBTB17 in thymocytes after β-selection.

### ZBTB16 (PLZF)

ZBTB16 (PLZF, promyelocytic leukemia zinc finger) is expressed lowly in early ETPs, slightly increased at the DN2 stage and then gradually decreased as cells progress toward DN3, DN4 and DP stages in the thymus (42, 43). It is a powerful TF that mainly regulates the development and function of innate-like unconventional T cells, including iNKT & γδ^+^ T cells, as well as ILCs both in mice and humans (**Figures 1A, B** **)** (44–47).

ZBTB16 drives the innate-like effector differentiation of CD1d-restriced iNKT cells. It is specifically and highly expressed in stage 1 (NK1.1^-^CD44^-^), and gradually declines thereafter in stage 2 (NK1.1^-^CD44^+^) and stage 3 (NK1.1^+^CD44^+^) iNKT cells. Deficiency of ZBTB16 has no impact on early thymic development of iNKT cells till stage 1, but almost completely abrogates their intrathymic expansion and effector differentiation that characterize their lineage (48, 49). Accordingly, ZBTB16-deficient iNKT cells retain a naive CD44^low^CD62L^high^ phenotype and accumulate in peripheral lymph nodes rather than the liver (48, 49), as ZBTB16 directly binds and regulates genes encoding cytokine receptors (IL-12R1, IL-18R1 & CCR4, etc) and homing/adhesion molecules (CD62L & CD44) in iNKT cells (46, 50). ZBTB16 cooperates with the TF YY1 (Yin Yang 1) to promote the development of iNKT cells beyond stage 2, and acquire the innate feature of “preformed” mRNAs that enable their rapid and simultaneous production of IFN-*γ* & IL-4 upon primary activation (51). Although ZBTB16 and YY1 do not regulate each other’s expression, they form a protein complex which appears to mediate some functions of ZBTB16 in iNKT cells. For instance, the ZBTB16/YY1 complex promotes the binding of ZBTB16 to target DNA sequence and thus augments its transcriptional activity (51). Alternatively, given that both ZBTB16 and YY1 can serve as chromatin remodelers, it is conceivable that one may function as a transducer to relax the chromatin, and thereby enhance the transcriptional activity of the other. Of note, studies have indicated that the protein level of ZBTB16 is important for iNKT cell development, as reduced ZBTB16 expression results in significantly decreased iNKT cells in the thymus and periphery, especially ZBTB16^high^ iNKT2 cells (52, 53). This dose effect of ZBTB16 in iNKT development and subset specification might be related to differences in the coverage of ZBTB16 binding sites in the genome, which subsequently generate different epigenomic landscapes and eventually lead to different gene expressions.

Apart from αβ^+^ iNKT cells, ZBTB16 is expressed in around 70% of thymic and 40% of peripheral V*γ*1^+^Vδ6.3^+^ *γ*δ T cells in adult mice as well as in V*γ*5^+^ & V*γ*6^+^ *γ*δ T cells in fetus thymus (43, 54, 55). Similar to what observed in iNKT cells, ablation of ZBTB16 mildly reduces the number of V*γ*1^+^Vδ6.3^+^ *γ*δ T cells, prevents their downregulation of lymph-homing molecule CD62L, and completely abrogates their ability to coexpress IFN-*γ* & IL-4 (54, 55). Interestingly, deficiency of ZBTB16 leads to a profound and specific impairment of thymic V*γ*6^+^ but not V*γ*5^+^ *γ*δ T-cell development, and moreover, ZBTB16-deficient CD27^+^CD44^-^ immature V*γ*6^+^ *γ*δ T cells fail to proliferate, which is necessary for them to acquire a mature CD27^-^CD44^+^ phenotype with increased IL-17-secreting ability (43). As overexpression of the anti-apoptotic protein BCL2 or depletion of the pro-apoptotic factor BCL2L11 (BCL2 like 11, commonly known as BIM) fails to revert the impaired development/function of iNKT or *γ*δ T cells in ZBTB16-deficient mice (42, 43, 52), it was suggested that ZBTB16 may promote the expansion and acquisition of effector phenotype of innate T cells in the thymus either cell-intrinsically or indirectly *via* facilitating their thymic retention (42, 44). In mature iNKT and *γ*δ T cells, ZBTB16 seems to augment their inflammatory functions through regulating the mitochondrial activity and glucose metabolism (56). In addition, ZBTB16 is also highly expressed in MAIT cells and unconventional CD8α^+^CD8β^-^TCRαβ^+^ T cells, both in mice and men (49, 57, 58). The innate-like unconventional CD8α^+^CD8β^-^TCRαβ^+^ T cells, which are NK1.1^+^ in mice or CD161^+^ in humans, are abundantly present in livers, exhibit an activated/memory phenotype, and use a perforin-dependent mechanism to dampen autoimmune responses *in vivo* (58). Not unexpectedly, the development of these MAIT and unconventional CD8α^+^CD8β^-^TCRαβ^+^ T cells are highly, albeit not absolutely, dependent on ZBTB16 as well (57–60).

In iNKT cells, TCR signals induce EGR2, which directly binds and subsequently transactivates *Zbtb16* (61). A recent study performed by Mao et al. indicated that, by binding to a critical regulatory intronic region in *Zbtb16* locus shared by iNKT, MAIT, *γ*δ T cells and ILCs, RUNX1 directly activates the expression of ZBTB16 and thus drives their development and effector differentiation (62). However, why ZBTB16 is only expressed in certain subsets of *γ*δ T cells with particular TCR repertoires (i.e., V*γ*1^+^Vδ6.3^+^ or V*γ*6^+^) remains obscure. In sharp contrast, ZBTB16 is silenced in conventional αβ^+^ T cells early in development and this repression appears to be stably maintained in their whole life span, regardless of the strength of TCR signaling and inflammatory conditions (63). Nevertheless, transgenic expression of ZBTB16 confers some innate-like memory/effector phenotypes and functions to conventional T cells, in both thymus and periphery, without a requirement for agonistic TCR signaling and cell proliferation (49, 64–66). Although these ZBTB16-expressing conventional αβ^+^ T cells bear similar TCR repertoire as control cells and do not upregulate NK markers, they phenotypically and functionally resemble normal Tm cells in terms of CD62L/CD44 expressions, cytokine profiles and sensitivities to TCR-mediated stimulation (64, 66). In addition to cell-intrinsic activity, ZBTB16 can also affect the phenotype/function of conventional T cells *in trans.* IL-4, secreted by the expanded population of ZBTB16-expressing innate cells in mice with transgenic MHC-II on thymocytes or mutations in TCR signaling molecules (*Itk* or *Id3)*, induces the generation of innate-like Eomes-expressing CD4^+^/CD8^+^ T cells as well as CD4^+^Foxp3^+^CD103^+^ Treg cells (44, 67).

Unlike its restricted expression in murine T cells (essentially in all iNKT but only a subset of *γ*δ^+^ T cells), ZBTB16 is expressed in virtually all *γ*δ^+^ T, NK and NKT cells as well as in a large fraction of CD161^high^ CD4^+^/CD8^+^ T cells in humans (47, 49). ZBTB16 seems to regulate the number, phenotype and/or function of these cells as the phenotype of iNKT cells is altered and the number of CD161^high^CD8^+^ T cells is reduced in an individual with a biallelic loss of functional ZBTB16 (47). Surprisingly, ZBTB16-expressing CD161^high^CD8^+^ T cells expressed less IFN-*γ* than their ZBTB16-negative CD161^-^CD8^+^ counterparts, and NK cells from the ZBTB16-disrupted individual seemed to produce more cytokines than the control samples (47). Moreover, the increased/decreased numbers of ZBTB16-expressing CD8^+^ T cells in patients with metastatic melanoma/autoimmune disease, respectively, suggest that these cells, akin to their murine CD8α^+^CD8β^-^TCRαβ^+^ counterparts, may be capable of suppressing the inflammatory responses *in vivo* (47, 58). Interestingly, a recent report revealed that ZBTB16 is specifically upregulated in human Th17 cells possibly by ROR*γ*t, as ROR*γ*t downregulation diminishes its expression. Upon expression, ZBTB16 not only directly binds to the promoters of *Ccr6* & *Rorc* and activates their expressions, but also collaborates with ROR*γ*t to induce the expression of many other Th17-associated genes (68). Notably, studies indicated that ZBTB16 predisposes human iNKT, MAIT, conventional T as well as Jurkat cells to apoptosis induced by FAS or staurosporine (a protein kinase C inhibitor), partially through directly inducing the expression/activation of Caspase 3 (69).

In sum, ZBTB16 represents an essential factor coordinating the development and effector function of murine and human innate T cells (**Figures 1A, B** **)**. In humans, it may regulate the survival and differentiation of conventional T cells as well (**Figure 2A**). ZBTB16 exerts these functions as a TF and/or a chromatin remodeler to activate/repress gene expressions in a context-dependent fashion.

## ZBTB Proteins Regulating Both the Development and Function of T Cells

### ZNF131 (ZBTB35)

ZNF131, also known as ZBTB35, is originally identified as a binding partner of ZBTB33 (KAISO). It contains a typical BTB domain and five or six ZF domains created by alternative mRNA splicing (70). The ZNF131/ZBTB33 heterodimer alters the transcriptional activity of each protein, and thus fine-tunes each-mediated biological process in cells (70, 71).

Ablation of ZNF131 in the DN2/DN3 thymocytes (Lck-Cre) does not affect *γ*δ^+^ T cell development, but significantly reduces the numbers of DN3, DN4 & DP cells, especially the latter two populations (72). Although the rearrangement of TCR-β locus is intact in ZNF131-deficient DN3 cells, ZNF131 represses the expression of CDKN1A (cyclin dependent kinase inhibitor 1A, also known as P21^Cip1^) by directly binding to its promoter region, and therefore promotes the (pre-)TCR-induced proliferation of thymic DN3/4 & peripheral mature T cells (**Figures 1A**, **2A, B**). Notably, PMAIP1 (Noxa) and BAX, two other TP53 target genes, are only upregulated in ZNF131-depleted thymocytes but not in mature T cells, indicating a differential involvement of TP53 activity among these cells. In addition, depletion of ZNF131 at the DP stage (CD4-Cre) has no impact on the survival & differentiation of DP toward CD4 or CD8 SP cells in the thymus. Thus, ZNF131 is mainly involved in the DN-to-DP transition of thymocytes and the homeostasis/proliferation of mature T cells *via* suppressing CDKN1A (72). Recently, the same group showed that ZNF131 promotes the early B cell development *via* similar mechanisms (73).

### ZBTB7B (THPOK) and ZBTB7A (LRF)

ZBTB7B, also called THPOK (T-helper-inducing POZ/Kruppel-like factor), controls CD4^+^ *vs.* CD8^+^ T-cell lineage commitment in the thymus (74). Although not required for the β-selection and survival of thymocytes, ZBTB7B is selectively upregulated, likely by persistent TCR signaling that overrides the silencer activity of its distal regulatory element (DRE), in MHC-II-restricted CD69^+^CD4^+^CD8^low^ intermediate thymocytes (INTs) where lineage is thought to be determined (74–78). Functionally, disrupted expression/function of ZBTB7B redirects MHC-II-restricted INTs into the CD8^+^ lineage, while its overexpression converts MHC-I-restricted INTs into the CD4^+^ lineage (**Figure 1A**) (75, 76, 79–82). Notably, it appears that ZBTB7B is neither absolutely necessary nor sufficient for the specification of CD4^+^ T-cell fate. Mice lacking both ZBTB7B and RUNX factors still generate CD4^+^ SP cells, and moreover, ZBTB7B fails to promote CD4^+^ T-cell differentiation in the absence of GATA3 (74, 76, 81). Rather, ZBTB7B regulates CD4^+^ *vs.* CD8^+^ T-cell lineage commitment in the thymus mainly by repressing RUNX3, which promotes CD8 while silences CD4 expression (74, 81, 82). The study performed by Luckey et al. indicated that ZBTB7B represses RUNX3 through transcriptionally activating *Socs1* & *Cish*, because it is unable to repress RUNX3 and prevent thymocytes from adopting the CD8^+^ lineage fate in the absence of SOCS1, and conversely, transgenic expression of SOCS1 in ZBTB7B-deficient mice largely replaces the latter’s role in inducing the generation of CD4^+^ T cells (82). However, a recent study revealed that ZBTB7B-depletion does not affect the expression of SOCS family genes in MHC-II-signaled immature MHC-I^low^CD44^low^ CD4^+^ SP thymocytes in RNA-Seq analysis (83), therefore challenging the ZBTB7B-SOCS1-RUNX3 model in thymocytes proposed by Luckey et al. (82). Additionally, ZBTB7B may be able to directly bind to and subsequently inactivate the silencer of *Cd4*, thereby derepressing/maintaining CD4 expression in developing/mature CD4^+^ T cells (74, 84).

By contrast, mice with an inducible deletion of ZBTB7A (LRF, leukemia/lymphoma-related factor) exhibit a grossly normal thymic T-cell development, but an aberrantly increased number of extrathymic DP T cells in the bone marrow (BM) that is reversed by blocking NOTCH signaling (**Figure 1A**) (85, 86). ZBTB7A-deficient B220^+^ pre-pro-B cells express high levels of NOTCH1 & 3, and readily differentiate into DP T cells in the presence of NOTCH ligand DLL1-expressing stromal cells (85). Moreover, ZBTB7A promotes the homeostasis of HSCs and prevents their pre-mature differentiation towards T cells by repressing the expression of DLL4 on erythroblasts (86). ZBTB7A thus prevents the pre-mature T-cell lineage commitment of developing HSCs by opposing NOTCH signaling, both intrinsically and extrinsically, in the BM (85). The inhibition of NOTCH signaling by ZBTB7A seems to be overruled by the abundantly expressed NOTCH ligands on thymic stromal cells to allow for T-cell development in the thymus, as no visible impact of depletion of ZBTB7A in DP thymocytes (CD4-Cre) on subsequent T-cell development and function is noted (87, 88). In addition, ZBTB7A promotes the erythroid differentiation in mice and humans (21).

Interestingly, despite the strikingly different roles of ZBTB7A & ZBTB7B in T-cell development, these two proteins act redundantly in maintaining the integrity and function of mature CD4^+^ T cells (**Figures 1A**, **2A, B**) (87, 88). ZBTB7A enables the “redirected” MHC-II restricted ZBTB7B-deficient CD4^-^CD8^+^ T cells to regain surface CD4 and CD40L upon activation (87), and prevents the transdifferentiation of CD4^+^ T cells into CD4^-^CD8^+^ T cells in *Zbtb7b* ^pd^ (*Zbtb7b* peripheral deleter) mice, in which the depletion of *Zbtb7b* mainly occurs in postthymic CD8^+^ SP and Foxp3^-^CD4^+^ SP T cells (Cre driven by the human CD2 promoter) (88). Although not required for Th17 differentiation, ZBTB7B promotes the *in vivo* differentiation of Th2, and prevents differentiated Th1/Th2 from diverging into Tc cells mainly by restraining the expression/activity of RUNX3, which silences CD4 & IL-4 whereas concomitantly activates the expression of cytotoxic molecules (i.e., CD8α, Eomes, perforin and granzyme B, etc) (88, 89). Given that ZBTB7A is as efficient as ZBTB7B at antagonizing RUNX-mediated *Cd4* silencing (88), it is likely that ZBTB7A partially compensates for the loss of ZBTB7B by repressing the activity of RUNX proteins to maintain the phenotype and residual Th functions of CD4^+^ T cells in *Zbtb7b* ^pd^ mice. Moreover, deletion of both ZBTB7A & ZBTB7B has a greater impact, than deletion of the latter only (all mediated by CD4-Cre), on the number/phenotype and function of thymic Treg cells (90). Accordingly, despite the little effect of single deficiency of either ZBTB7A or ZBTB7B (Foxp3-Cre) on peripheral Treg cells, double knockout of both genes in Treg cells leads to a lethal inflammatory disease similar to that in Scurfy mice lacking functional CD4^+^Foxp3^+^ Treg cells. Notably, the redundant function of ZBTB7A and ZBTB7B in promoting the *in vivo* survival/fitness of effector Treg cells is independent of RUNX3 repression, but may be attributed to their functional overlap in supporting IL-2/Foxp3-mediated gene expressions (90). Nonetheless, ZBTB7B seems to nonredundantly stabilize Foxp3 and augment the suppressive function of Treg cells, especially in the mucosal-environment interface (90–92). In addition, a recent study by Vacchio et al. showed that inactivation of ZBTB7B in mature CD4^+^ T cells (CD2-Cre) almost completely blocks the differentiation of Tfh cells and the production of antigen-specific IgG *in vivo* (93). Mechanistically, ZBTB7B directly binds to regulatory regions of *Bcl6* and *Maf* to upregulate their expressions, and thus forced expressions of the latter two proteins cooperatively promote the differentiation of ZBTB7B-deficient T cells towards Tfh cells (93).

Given the role of ZBTB7B in regulating the lineage choice into CD4^+^ or CD8^+^ T cells in the thymus (74), and that its ectopic expression in mature CD8^+^ T cells leads to loss of some CD8^+^ T-cell characteristics while gain of many CD4^+^ T-cell features (94), silencing of ZBTB7B was thought to be irreversible in peripheral CD8^+^ T cells to maintain their integrity & function. Unexpectedly, ZBTB7B repression is readily abrogated in mature CD8^+^ T cells upon TCR stimulation through the proximal enhancer & promoter at its locus (77, 95). Although such a low level of ZBTB7B (~ one-tenth of that in CD4^+^ Tn cells) has little impact on the phenotype of CD8^+^ T cells *in vivo*, its functional deficiency not only significantly inhibits the clonal expansion of CD8^+^ T cells, but also greatly reduces the production of IL-2 and granzyme B in long-lived CD8^+^ Tm cells upon rechallenge, possibly *via* transcriptionally activating genes involved in DNA repair and repressing CD160, an exhaustion-associated molecule (95). In line with its role in promoting memory responses, a recent work from Bosselut’s group showed that ZBTB7B is required early during activation to safeguard the transcriptome signature and memory potential of CD4^+^ T cells as well (96). Moreover, ZBTB7B cell-intrinsically represses a dysfunctional and effector-like transcriptional program in CD4^+^ Tm cells by directly antagonizing the expression/activity of RUNX3 & PRDM1 (Blimp1) (96). ZBTB7B hence functions as an important regulator promoting the generation and functional fitness of both CD4^+^ and CD8^+^ Tm cells (**Figures 2A, B** **)**.

Functions of ZBTB7B in T-cell compartment extend beyond conventional αβ^+^ T cells as it is also expressed in a subset of *γ*δ^+^ DN thymocytes and essentially in all Vα14 iNKT cells (55, 97–100). Although dispensable for the early development, survival and proliferation of *γ*δ^+^ thymocytes, deficiency/overexpression of ZBTB7B reduces/increases the number of mature CD24^-^ *γ*δ^+^ (particular NK1.1^+^CD24^-^ *γ*δ^+^) thymocytes, respectively (**Figure 1A**) (99, 100). Likewise, disruption of ZBTB7B does not impact the number of total iNKT cells in mice, but significantly induces more IL-17-producing iNKT17 cells at the expense of IL-4-producing iNKT2 cells (**Figure 1B**). Additionally, depletion of ZBTB7B results in the appearance of atypical CD4^-^CD8^+^ iNKT cells, concurrent with the absence of the CD4^+^CD8^-^ subset (97, 98, 101, 102). Given the low/high levels of ZBTB7B in iNKT17/iNKT2 cells, and that enforced ZBTB7B expression represses ROR*γ*t, it appears that ZBTB7B fine-tunes the subset differentiation of iNKT cells (101, 102). Similar to that in conventional CD4^+^ T cells, ZBTB7B functions downstream of GATA3 in regulating the differentiation/function of iNKT cells (76, 97).

As outlined above, ZBTB7B represents a critical factor, sometimes partially redundant with ZBTB7A, that regulates the development, differentiation, homeostasis and function of both conventional αβ^+^ T and unconventional (*γ*δ^+^ and iNKT) T cells in mice. In humans, ZBTB7B seems to limit RUNX3-induced cytotoxic potentials of differentiated Th1 cells as well (103). Further investigations are needed to identify the network regulating protein levels of ZBTB7A & ZBTB7B and to dissect the molecular mechanisms responsible for their stage/context-dependent effects along the T-cell development/differentiation program.

### PATZ1 (ZBTB19 or MAZR)

PATZ1 (POZ/BTB and AT hook containing ZF 1), also known ZBTB19 or MAZR (MYC-associated ZF related-factor), is broadly expressed in multiple hematopoietic lineages. Germline deficiency of PATZ1 results in premature death of mice due to defects in the cardiac and neural systems (104).

PATZ1 is part of the molecular machinery that represses CD8, ZBTB7B and CD4 in developing thymocytes and/or mature CD8^+^ T cells. It is highly expressed in DN thymocytes, and then gradually declines along their progression to DP and subsequent CD4^+^/CD8^+^ SP stage (105). PATZ1 binds to multiple *Cd8* enhancer regions and prevents the premature expression of CD8 during DN to DP transition (**Figure 1A**), partially by recruiting NCoR *via* its BTB domain to condense the chromatin (105, 106). Later, it participates in CD4 *vs.* CD8 lineage commitment of MHC-I-signaled DP thymocytes by directly repressing ZBTB7B to restrain the transdifferentiation of developing CD8^+^ SP into CD4^+^ thymocytes (106). Moreover, PATZ1 physically interacts with RUNX1/RUNX3 *via* its 7^th^ ZF, and acts synergistically with RUNX1 in unsignaled TCRβ^low^CD69^-^ DP thymocytes or RUNX3 in mature CD8^+^ T cells to repress ZBTB7B (**Figure 1A**). Accordingly, PATZ1/RUNX1 or PATZ1/RUNX3 double-mutant mice exhibit increased expression of CD4 in both DN thymocytes and peripheral CD8^+^ T cells than RUNX1 or RUNX3 single-mutant mice, respectively (107). Given that a role of PATZ1 in silencing *Cd4* is only evident in cells with disrupted RUNX proteins, whereas it is capable of repressing ZBTB7B in RUNX-sufficient mature CD8^+^ T cells, there seems to be locus-specific differences in PATZ1 and/or RUNX proteins-mediated repressions of target genes in these cells.

In addition, PATZ1 regulates the iNKT cell subset differentiation. T-cell specific depletion of PATZ1 (Lck-Cre) results in an increased number of IL-4-producing iNKT2 cells at the expense of iNKT1 and iNKT17 cells (**Figure 1B**), but the total number of iNKT cells as well as their survival & proliferation is not affected (108). In line with the role PATZ1 in antagonizing ZBTB7B and CD4 expressions in conventional T cells (106, 107), PATZ1-deficient iNKT cells express increased levels of CD4 and ZBTB7B, and moreover, the combined loss of PATZ1 & RUNX3 results in a further increase of ZBTB7B, accompanied with enlarged/reduced proportions of iNKT2/iNKT17 cells, respectively (108). Additionally, PATZ1-deficient iNKT cells express more EGR2 (108), which controls iNKT lineage differentiation and is normally expressed at the highest level in iNKT2 cells, followed by iNKT17 and iNKT1 cells (61, 109). Considering the aforementioned roles of ZBTB7B in iNKT development (97, 98, 101, 102), it seems that PATZ1 regulates the subset differentiation of iNKT cells mainly by fine-tuning expressions of ZBTB7B and EGR2. Nonetheless, whether these two regulatory processes are connected or totally independent during iNKT development remains to be determined.

Two recent studies from the same group showed that PATZ1 is capable of regulating the development of Treg cells and the generation of CD8^+^ Te/Tm cells independent of ZBTB7B (**Figures 1A**, **2A, B**) (110, 111). Levels of PATZ1 progressively decline during the development/differentiation of both murine and human Foxp3^+^ Treg cells, suggesting that it may negatively regulate Treg development. Indeed, specific depletion of PATZ1 in T cells (CD4-Cre) results in an increased percentage/number of Treg cells, while its enforced expression dose-dependently impairs the development of thymic Treg cells as well as the differentiation of induced Treg (iTreg) cells in the periphery (110). Interestingly, comparable levels of PATZ1 in Foxp3^+^ *vs.* Foxp3^-^ cells were observed in culture with TGF-β, and PATZ1-sufficient or deficient Treg cells display minor transcriptional differences, indicating that PATZ1 alone is neither decisive for the induction of Foxp3 nor essential in establishing/maintaining the transcriptional signature of Treg cells. Nonetheless, PATZ1-deficient Treg cells show a slightly increased Foxp3 stability and suppressive capacity *in vivo*, but display a significantly reduced survival/proliferation after transfer into RAG2-deficient recipient mice (110). In CD8^+^ T-cell compartment, albeit the little effect of PATZ1-deficiency alone, combined depletions of both PATZ1 and RUNX3 lead to a much more pronounced downregulation of Tc signature genes (*Gzmb*, *Tbx21* and *Cd8a*, etc) as compared to the single RUNX3 deletion, indicating that PATZ1 partially compensates for the loss of RUNX3 in the differentiation of CD8^+^ Te cells (111). In contrast, PATZ1 and RUNX3 exert distinct functions in the formation of CD8^+^ Tm cells. Depletion of RUNX3 greatly impairs the differentiation of Tm, whereas ablation of PATZ1 promotes the generation of CD62L^-^CD127^+^ effector memory T (Tem) at the expense of CD62L^+^CD127^+^ central memory (Tcm) CD8^+^ T cells (111). Thus, PATZ1 appears to team up with RUNX3 in promoting the naive-to-effector transition of CD8^+^ T cells, while at later stages it negatively regulates the generation of Tem cells independent of RUNX3, presumably in part through regulating the Tem-*vs.*-Tcm diversification process (111). Notably, PATZ1 regulates the differentiation of Treg and CD8^+^ Tc cells independent of ZBTB7B, as its deficiency does not affect ZBTB7B expression in CD4^+^ T cells (110), and similar effects were observed in CD8^+^ T cells depleted with ZBTB7B (111).

Given that PATZ1 may, based on the cellular context, promote/inhibit the survival & proliferation of cells in a TP53-dependent/independent manner (112, 113), the relative contributions of these mechanisms to its roles in iNKT and conventional T cells remain to be investigated.

## ZBTB Proteins Mainly Affecting the Function of T Cells

### BCL6 (ZBTB27)

The well-studied BCL6 (B cell lymphoma-6, also known as ZBTB27) was initially identified as an oncogene frequently translocated/hypermutated in diffuse B cell lymphoma (DLBCL) and follicular lymphoma (FL) cells (22). In T-cell compartment, BCL6 not only regulates the differentiation of Th cells, but also impacts the generation & maintenance of Tm cells (**Figures 2A, B** **)** (114, 115).

BCL6 is indispensable for germinal center (GC) reactions as it drives the differentiation of both GC B cells and Tfh cells (22, 114). BCL6 is induced as early as the first division after T-cell activation in a CD28-dependent manner. CD28 signal quickly upregulates the H3K36 methyltransferase NSD2 (nuclear receptor binding SET domain protein 2), which specifically modifies the gene body of *Bcl6* and hence enhances its transcription (116). Thereafter, multiple TFs and signaling pathways are employed to enhance/maintain BCL6 levels (114). For instance, by binding to IL-6R whose expression is mediated by TCF7, IL-6 enhances the expression BCL6 in CD4^+^ T cells *via* STAT1/3, and thus promotes the differentiation of Tfh cells both *in vitro* and *in vivo* (117–119). Subsequently, BCL6 directly binds and represses a set of TFs (*Prdm1*, *Tbx21*, *Gata3, Rora/Rorc* and *Runx2/3*, etc), signaling transducers (*Stat5*), cytokines (*Il4* & *Il17a*) and chemokine receptors (*Selplg, Ccr7* and *Gpr183*, etc) that drives Th cells towards non-Tfh fates/functions. At the meantime, BCL6 indirectly upregulates many essential functional molecules, such as CXCR5, ICOS, PD1 and IL-6R, likely by repression-of-repressor mechanisms through targeting a panel of TFs including *Prdm1*, *Id2*, *Runx2/3* and *Klf2* (114). An extensive review on the regulation of BCL6 expression as well as BCL6-mediated transcriptional regulation in Tfh cells has just been published by Choi and Crotty elsewhere (114).

Misawa et al. recently revealed that sequestration of BCL6 in the cytoplasm represents another layer to fine-tune its function in Tfh cells (120). PRKD2 (protein kinase D2) directly binds and phosphorylates BCL6, which reduces its nuclear translocation in resting CD4^+^ T cells. After entering the nucleus, BCL6 represses *Prkd2* transcription, and thereby irreversibly drives Tfh differentiation & function (120). Apart from differentiation, BCL6 is also essential for the maintenance of Tfh cells *in vivo*, as temporal ablation of BCL6 by tamoxifen (CD4-Cre^ERT2^) induces the transdifferentiation of established Tfh cells into TBX21^+^ Th1 cells during acute viral infection (121).

Intriguingly, although BCL6 antagonizes the differentiation of non-Tfh subsets (Th1, Th2 and Th17, etc) by directly repressing their lineage-defining TFs (*Tbx21*, *Gata3* and *Rorc*, etc) as mentioned above (114), it is lowly expressed in these Th subsets and modulates their functions *via* multiple mechanisms (**Figure 2A**). In Th1 cells, BCL6 is guided by TBX21, *via* the TBX21-BCL6 complex which masks BCL6’s DNA binding ZF domains, to promoter regions and represses the transcriptional activation of *Socs1/3*, *Tcf7* and *Ifng*, whereas excess amounts of BCL6 induce Tfh gene expressions (122, 123). Therefore, lowly expressed BCL6, by cooperating with TBX21, not only promotes the identity and function of Th1 cells, but also restrains the overproduction of IFN-*γ* to prevent possible collateral tissue damages *in vivo*. Likewise, it directly targets and dampens the ability of GATA3 to transactivate Th2 genes in Treg cells, and concomitantly strengthens their ability to control allergic airway inflammations (124, 125). Moreover, BCL6 upregulates CXCR5 in Foxp3^+^ Treg cells, and thereby endows these so-called T follicular regulatory (Tfr) cells with the capacity to migrate towards the B cell follicular to limit the magnitude of GC responses (126, 127). In addition, BCL6 maintains Foxp3 expression and promotes the suppressive ability of tumor-infiltrating Treg cells, and thus high BCL6 levels in Treg cells represent a poor prognosis marker in patients with colorectal cancer & melanoma (128). Of note, how BCL6 maintains the lineage stability and functional fitness of Treg cells specifically in tumor microenvironment remains unsolved. In Th9 cells, BCL6 represses STAT5-mediated transactivation of *Il9* by competing for binding motifs in *Il9*’s promoter region (129, 130).

Although BCL6 has little effect on the initial proliferation of CD8^+^ Tn cells, it not only promotes the generation of CD8^+^ Tcm cells at the effector/precursor stage through, at least partially, upregulating the expression of TCF7 (131, 132), but also amplifies their secondary expansion (**Figure 2B**) (131, 133). Likewise, BCL6 promotes the generation of long-term CD4^+^ Tm cells by protecting their precursors from undergoing apoptosis (**Figure 2A**) (134). Moreover, BCL6 binds to the promoter region of *Gzmb* and represses its expression in CD8^+^ Te cells (135). A recent study disclosed that BCL6 directly represses a panel of genes involved in the glycolysis pathway, including *Slc2a1/3*, *Pkm* & *Hk2*, in Th1 cells exposed to low levels of IL-2 (136). Given that the differentiation and function of effector/memory T cell is coupled with metabolic reprogramming, and that inhibiting glycolysis favors the generation of CD8^+^ Tm cells (19), it is conceivable that BCL6 may regulate the effector-*versus*-memory fate decision of T cells as well as their proliferation & function *via* impacting metabolic pathways as well.

Two recent studies extended roles of BCL6 in T-cell compartment from the periphery (differentiation and function) to thymus (early development) (**Figures 1A, B** **)** (137, 138). Pre-TCR signal upregulates BCL6 in DN3 to DP thymocytes, and depletion of BCL6 from the DN2 stage onwards (Lck-Cre) results in a reduced proliferative burst of β-selected DN3 cells, impaired survival of DN4 thymocytes, and an aberrantly augmented NOTCH1 activation in DP thymocytes (138). Given the ineffectiveness of BCL6-ablation in DP stage (CD4-Cre) on subsequent T-cell development (137), BCL6 seems to specifically promote the differentiation of DN3 to DP stage of conventional αβ^+^ T cells (138). By contrast, depletion of BCL6 in DP thymocytes greatly impairs the development of iNKT and MAIT cells (137). BCL6 is transiently expressed in CD24^high^ iNKT cells (stage 0), and is required for their subsequent transition into stage 1 (CD24^low^). Despite the normal proliferation and survival, BCL6-deficient iNKT cells fail to shape the chromatin accessibility and upregulate genes, such as *Zbtb16*, required for innate-like T-cell development & function (137). Given the essential role of ZBTB16 in other innate T cells like *γ*δ^+^ T cells, BCL6 may regulate their development and function through ZBTB16 as well.

### BCL6B (ZBTB28)

BCL6B, also known as ZBTB28 or BAZF (BCL6-associated ZF protein) is 65%/94% identical at the BTB/ZF domain to that of its most closely related homologue BCL6, respectively (139). As such, these two proteins not only bind to the same target sequence, but also may form heterodimers *via* their BTB domain & the middle portion to cooperatively regulate target gene expression (140).

Mice with germline BCL6B-deficiency are grossly normal and display no overt abnormalities in thymic T-cell development (141, 142). BCL6B is rapidly upregulated in T cells after activation, and highly expressed in CD4^+^ and CD8^+^ Tm cells. Although BCL6B seems to specifically promote TCR-triggered proliferation of CD4^+^ Tn cells *in vitro* (**Figure 2A**) (142), BCL6B-deficient mice exhibit no defects in CD4^+^ T-cell priming and T-cell dependent antibody productions *in vivo* (141). Moreover, the absence of BCL6B has no impact on the primary proliferation of CD8^+^ T cells as well as the generation and maintenance of CD8^+^ Tm cells. However, BCL6B-ablated CD8^+^ Tm cells express little IL-2 and fail to undergo a robust proliferation in the secondary response (**Figure 2B**) (141).

It has been reported that the BTB domain of BCL6B can not recruit the BCoR or NCoR complex by itself, hence BCL6B needs to associate with BCL6 to exert its repressive function (140). Given the same DNA binding sequence in BCL6 & BCL6B and that TBX21/BCL6 complex masks BCL6’s DNA-binding motif (122, 123, 140), it is conceivable that BCL6B may compete with TBX21 to dimerize with BCL6 and thereby augment its repression of BCL6-, but not TBX21-, target genes in cells with limited amount of BCL6. In this scenario, the reduced IL-2-producing ability and secondary expansion of BCL6B-depleted CD8^+^ Tm cells may just be a reminiscent of the impaired generation and expansion of CD8^+^ Tcm cells resulting from BCL6 deficiency.

### HIC1 (ZBTB29)

HIC1 (hypermethylated in cancer 1), is a member of ZBTB family (ZBTB29) that has been extensively studied in cancer. HIC1 mainly acts as a tumor suppressor that regulates cellular chromosome stability, survival and proliferation of various tumors *via* TP53-dependent or independent mechanisms (143). Recent studies identified HIC1 as an essential TF regulating the intestinal immune homeostasis *via* αβ^+^, *γ*δ^+^ T cells and group 3 ILCs (**Figures 1A**, **2A, B**) (144–146).

By using fluorescence-reporter mice (HIC1^Citrine^), Burrows et al. found that HIC1 is only expressed in intestinal immune cells, including T cells, dendritic cells and macrophages, but not B cells (146). Consistent with this restricted expression pattern, HIC1 seems to be only induced in T cells during activation in the presence of vitamin A metabolite, retinoic acid, which is abundantly present in intestines. Moreover, specific depletion of HIC1 in T cells (CD4-Cre) has no effect on thymic αβ^+^ T-cell development as well as their phenotypes & numbers in peripheral organs except the intestine (146). Deficiency of HIC1 significantly reduces the number of CD4^+^ & CD8^+^ T cells in intestinal lamina propria (LP) and intraepithelial (IE) compartments (partially) by reducing surface expressions of CD69 & CD103, two molecules mediating the retention of tissue-resident cells (146). HIC1 is not involved in the differentiation of Th1, Th2 and Treg cells, but binds to STAT3 and thereby inhibits the latter’s DNA binding and transcriptional activation of *Il17a*. Functionally, depletion/overexpression of HIC1 results in significantly increased/decreased productions of IL-17A in Th cells, respectively. Intriguingly, in spite of the augmented IL-17A-producing ability, HIC1-deficient CD4^+^ T cells are unable to elicit intestinal inflammation, but rather to ameliorate intestinal tissue damages induced by innate immune cells (146). Given that HIC1 directly represses SIRT1, which promotes T-cell tolerance/quiescence by regulating the activity of AP-1 or fatty acid oxidation (146–148), the upregulated SIRT1 expression/activity may account for the tolerogenic/dormant phenotype of HIC1-deficient CD4^+^ Th cells *in vivo*.

In humans, HIC1 precedes the expression of FOXP3 in iTreg cells differentiated by IL-2, TGF-β and all-trans retinoic acid (144). Although no effect on FOXP3 expression, knockdown of HIC1 results in a downregulation of the majority of iTreg signature genes (*Tigit* & *Ctla4*, etc), and a concomitant upregulation of genes associated with effector function/signaling (*Ciita*, *Tbx21* & *Gata3*, etc), indicating that HIC1-deficiency alters the transcriptome of iTreg cells and prepares cells for effector-like functions. Functionally, HIC1-deficient iTreg cells are less suppressive but produce more IFN-*γ* upon reactivation (144). Therefore, HIC1 appears to represent an important TF that programs and stabilizes human iTreg cells in intestines with plenty of retinoic acid (149).

Recently it was found that depletion of HIC1 in hematopoietic cells (Vav-Cre) significantly reduces the number of *γ*δ^+^ T cells and IL-22-producing ROR*γ*t^+^ group 3 ILCs, thereby rendering mice more susceptible to intestinal infections (145). As no differences on ILCs-precursors were observed between control and HIC1-deficient mice, HIC1 seems to be required for the homeostasis of group 3 ILCs in the intestine (145). Nonetheless, the underlying mechanisms as well as the role of HIC1 in the development/homeostasis and function of *γ*δ^+^ T cells remain unknown.

### ZBTB32

ZBTB32, originally named as FAZF (fanconi anemia zinc finger) due to its interaction with FANCC (fanconi anemia group C protein), displays a high homology with ZBTB16 (150). ZBTB32 is dispensable for thymic T-cell development and the survival of mature T cells as no significant differences in T cell compartment were observed among control, ZBTB32-deficient and -transgenic mice (151–153). Despite the low level of ZBTB32 in resting T cells, it is rapidly upregulated by NF-ATc2 in Th cells upon TCR-stimulation, and functions as a brake for T-cell activation/proliferation by attenuating TCR signaling and inhibiting the binding of NF-*κ*B to *Il2*’s promoter (**Figures 2A, B** **)** (151–153). In line with *in vitro* findings showing that ZBTB32 binds to GATA3 and represses the latter’s ability to transactivate target Th2 cytokine genes, ZBTB32-deficient/transgenic mice exhibit enhanced/reduced Th2 responses *in vivo*, respectively (154–156). By contrast, comparable experimental autoimmune encephalomyelitis (EAE) developments or diabetes incidences were observed between control and ZBTB32-deficient mice on the C57BL/6 or NOD background, respectively (153, 157–159). These results indicate that other genes, in some contexts such as the genetic background of mice and/or type/duration of inflammations, may compensate for the loss of ZBTB32 in CD4^+^ Th1/17 cells *in vivo*.

Interestingly, ZBTB32 represses the expression of IL-4 in CD8^+^ Tc2 cells independent of GATA3, but through recruiting HDAC1 & HDAC2 to maintain a repressive chromatin state of the *Il-4* locus (160). Depletion of ZBTB32 results in a hyper-activation and -proliferation of CD8^+^ T cells *in vitro* (152). Accordingly, ZBTB32-deficiency in CD8^+^ T cells significantly augments the magnitude of effector responses and the generation of antigen-specific CD8^+^ Tm cells in virus-infected mice, while its persistent expression exhibits an opposite effect (152, 161). Mechanistically, ZBTB32 represses the expression of CD27 and Eomes, two molecules known to promote the persistence and survival of long-lived CD8^+^ Tm cells, *via* collaborating with PRDM1, HDAC1 & HDAC2. ZBTB32 and PRDM1 bind together on the regulatory regions of *Cd27* & *Eomes*, and in the absence of ZBTB32, PRDM1 is unable to suppress their expressions. Likewise, the aberrantly increased ZBTB32, resulting from the loss of PRDM1, fails to prevent the overexpansion of effector and memory CD8^+^ T cells at the late stage of anti-viral responses either (161). Together, these data indicate that ZBTB32 is a key TF that not only limits the effector CD8^+^ T cell response, likely *via* inhibiting TCR signaling & NF-κB activity, but also cooperates with PRDM1 to restrain the generation of memory responses *via* repressing CD27 & Eomes (**Figure 2B**) (151, 153, 161).

Notably, given that ZBTB32-deficient CD8^+^ T cells display no defects in cytolytic functions, proliferate more robustly upon secondary challenge, and are more refractory to clonal exhaustion during persistent antigen stimulations (153, 161), inhibiting its activity/expression may enhance the efficacy of CAR-T-based immunotherapy in cancer patients. In addition, considering ZBTB32-deficient memory B cells exhibit a cell-intrinsic survival advantage owing to increased levels of proteins promoting the cell-cycle progression (E2F3 & PCNA) and mitochondrial function (MRPL-18/22/51) (162), whether these mechanisms hold true in T cells warrant further investigations.

### ZBTB24

Loss-of-function mutations of *ZBTB24* result in Immunodeficiency, Centromeric instability, and Facial anomalies syndrome 2 (ICF2), a rare autosomal recessive disease with main immunological characteristics of hypogammaglobulinemia and respiratory/gastrointestinal infections (163–165). Most ICF2 patients harbor *ZBTB24* nonsense mutations that generate premature stop codons, and the rest have missense mutations affecting key residues in the ZF domain that largely abrogate its DNA-binding capacity and transcriptional activity (164, 166). Despite the normal amounts of circulating T & B cells in most ICF2 patients upon diagnosis, detailed immunological phenotyping has revealed a lack of CD19^+^CD27^+^ memory B cells, a progressive depletion of B & CD4^+^ Th cells and an inverted CD4/CD8 ratio in circulations of ICF2 patients (167, 168). Apart from infections, autoimmune manifestations, inflammatory bowel diseases and malignancies have all been reported in ICF2 patients (169), arguing a dysregulated T-cell function *in vivo.*

Indeed, the antigen-stimulated expansion of peripheral T cells are significantly reduced in most ICF2 cases tested (164, 165, 170), possibly stemming from chromosome separation defects and/or increased cellular apoptosis (165, 171). In human T cells, knockdown of ZBTB24 demethylates the promoter region of *TNFRSF10A* (encoding TRAIL receptor 1) likely *via* repressing CDCA7 (cell division cycle associated 7) (171). CDCA7 binds to chromatin, recruits HELLS (helicase, lymphoid specific) and remodels nucleosomes to facilitate DNMT3B-mediated DNA methylation (172). ZBTB24-downregulated primary CD4^+^ T cells express increased levels of surface TRAIL receptor 1 and are more susceptible to TRAIL-induced apoptosis (171). Moreover, CHIP-Seq followed by ingenuity pathway analysis (IPA) showed an enriched binding of ZBTB24 to the promoters of genes involved in Th1 and Th2 activation pathways and cellular metabolic processes (173), although ZBTB24 neither heterodimerizes with nor affects the expression/transcriptional activity of BCL6 (174). Apart from functioning as a TF that directly transactivates target genes or as a chromatin remodeler by cooperating with CDCA7/HELLS & DNMT3B (173, 175), ZBTB24 directly interacts with PARP1 through its ZF domains, and is guided to DNA breaks (23). This interaction prolongs the stability of PARP1 at DNA damage sites, facilitates the assembly of LIG4/XRCC4 complex, and thus promotes error-free nonhomologous end-joining (NHEJ) and class-switch recombination (23). Intriguingly, the repertoires of BCR and TCR seem to be normal in ICF2 patients (23, 165), indicating that developing T/B cells employ other mechanisms to compensate for the lack of ZBTB24 during the VDJ recombination process.

Collectively, although ZBTB24 is dispensable for thymic T-cell development, the genotype-phenotype association study in ZBTB24-disrupted ICF2 patients and *in vitro* functional analyses demonstrated a cell-autonomous role of ZBTB24 in the survival, proliferation and function of T cells in humans (**Figure 2A**). Surprisingly, total body knockout of *Zbtb24* is embryonic lethal in mice (176), thus mice with an inducible or cell/stage-specific deletion of *Zbtb24* are needed to address its role in T cells *in vivo*.

### ZBTB20

Recently, mutations of *ZBTB20*, most of which result in amino acid substitutions in distal regions of its ZF motifs, were discovered to be responsible for primrose syndrome with multisystem failures (177). ZBTB20 is widely expressed in hematopoietic tissues, and promotes the long-term antibody responses by increasing the survival of BM plasma cells (22). ZBTB20 is transiently upregulated in activated CD8^+^ T cells, and in the steady state, its levels in CD8^+^ T-cell compartment are CD62L^+^CD44^+^ Tcm > CD62L^-^CD44^+^ Tem ≈ CD62L^+^CD44^-^ Tn > CD62L^-^CD44^-^ Te cells (178). Depletion of ZBTB20 in activated CD8^+^ Te cells, mediated by *Gzmb*-Cre, enhances the cellular glycolytic and mitochondrial metabolisms, and increases their mitochondrial fuel flexibility. Importantly, ZBTB20-deficiency renders CD8^+^ T cells, both phenotypically and transcriptionally, toward memory cells with an enhanced ability to mount secondary responses (178). Thus, ZBTB20 represents an important regulator that restrains CD8^+^ T cell metabolism and Tm differentiation in mice (**Figure 2B**). Detailed phenotypic and functional analyses on T cells in patients with dysfunctional ZBTB20 will shed more light on its role in human T cells.

### ZBTB33 (KAISO)

Despite the diverse roles of ZBTB33 (KAISO) in cell-cycle progression, genome stability and tumor invasion, ZBTB33-deficient mice are grossly normal, and exhibit comparable composition and phenotype of peripheral leukocytes as control mice (179–181). ZBTB33 binds to a motif in the *Ctse* locus in a methylation-dependent manner, and subsequently represses the expression of Cathepsin E and IL-10 in murine thymoma EL-4 cells, and in CD4^+^ T cells of MRL/lpr mice or patients with systemic lupus erythematosus (SLE) (182). In addition to the nucleus, ZBTB33 also localizes in the cytosol and membrane of cells, where it interacts with RhoH (Ras homolog family member H) and regulates chemokine-induced cytoskeletal reorganization and migration of both human Jurkat and murine splenic CD4^+^ T cells (**Figure 2B**) (183). Interestingly, RhoH seems to mediate the nuclear translocation of ZBTB33 in T cells following chemokine or TCR stimulation, but the functional consequence remains unknown (183).

## ZBTB Proteins in T-Cell Malignancy

Since the discovery of chromosomal translocations of ZBTB16 in human acute promyelocytic leukemia and BCL6 in B-cell lymphomas in the 1990s, numerous studies have been performed to investigate roles of ZBTB proteins in the development, function and neoplastic transformation of hematopoietic cells (21, 26, 184). Apart from modulating the expression/activity of TP53 and BCL2 in developing thymocytes as discussed above, ZBTB17 may promote the cell-cycle progression or malignant transformation of T cells by interacting with BCL6, GFI-1 and/or MYC (185, 186). Consequently, disrupting the interaction between ZBTB17 and MYC may render current acute lymphoblastic leukemia (ALL) chemotherapies more effective (187). Despite barely detectable in normal thymic CD3^+^ T cells, ZBTB7A is highly expressed in a subset of human T-cell lymphoma, and its overexpression induces the development of a mouse precursor T-cell lymphoma by directly repressing the transcription of the tumor suppressor gene *Cdkn2a* (*Arf*) (188). Likewise, constitutive expression of ZBTB7B during thymopoiesis propagates a preleukemic and self-renewing DN4 lymphoma progenitor population with mutations leading to enhanced NOTCH1 signaling and/or repressed tumor suppressors IKZF1 (Ikaros) & PTEN, which gives rise to T-cell lymphomas in the periphery (189). Moreover, a great proportion of human angioimmunoblastic T-cell lymphoma (AITL) express ZBTB7B, which appears to correlate with poor prognosis (190). ZBTB1 seems to specifically regulate asparagine synthesis in many T-lineage ALL (T-ALL) cell lines (like CUTTL-1, Jurkat & SUPT-1) by directly promoting the transcription of ASNS (asparagine synthetase), and thus may represent a potential target in the treatment of patients with T-ALL (191).

The malignant cell lines with abnormal expressions/activities of ZBTB proteins are excellent model cells for uncovering upstream signaling pathways/molecules regulating their expressions, and for deciphering molecular mechanisms underlying their roles in regulating the genome integrity and/or cell cycle progression, such as identifying target genes, interacting partners and key residues/motifs mediating these functions. These mechanistic studies not only further our understanding on their roles in hematopoietic cells, but also may inspire the design and test of novel agents with the ability to interfere the expression/function of ZBTB proteins. For example, given the essential role of BCL6 BTB domain in recruiting SMRT, NCoR and BCoR corepressors, small cell-penetrating peptide or compounds targeting the BCL6-BTB lateral groove pocket have been designed/identified, which are capable of killing B-cell lymphomas by inhibiting BCL6’s repressor function (192, 193). Nevertheless, their *in vivo* effect on lymphomas as well as on the function of primary lymphocytes needs further investigations.

## Concluding Remarks

As outlined above, ZBTB proteins play a fundamental and indispensable role in almost every aspect of T cell biology. They promote the survival & proliferation of early developing thymocytes, control the CD4 *vs.* CD8 lineage choice of conventional T cells, coordinate the development and effector function of innate-like T cells, and regulate the differentiation and function of mature Th and Tc cells. Although most of the aforementioned roles of ZBTB proteins in T cell biology are nonredundant, redundant functions of ZBTB7A and ZBTB7B in maintaining the integrity and effector potential of mature CD4^+^ T cells have been observed (87, 88). Despite that the ZF motifs bind to DNA and thus determine the transcriptional specificity of ZBTB proteins, they may regulate gene transcription in a cell type/subset specific fashion. It has been reported that binding motifs of BCL6 vary substantially among primary Th, macrophages and B cells (24). This context-dependent binding of BCL6 to genome sequence is likely attributed to the presence of interacting partners, such as TBX21 in Th1 cells, that mask BCL6’s DNA-binding ZF motifs, and/or different levels of proteins, such as STAT5 in Tfh and Th9 cells, capable of competing for bindings to the same DNA region (24, 129, 130). Apart from bindings to specific DNA sequences, interactions with different partners through the BTB or ZF domains of ZBTB proteins greatly impact their functions. For example, ZBTB17 regulates early T-cell development in a MYC-independent manner, while its role in T lymphoid malignancy is largely MYC-dependent (185).

Thus, new studies are required to further delineate the molecular mechanisms responsible for the subset- and stage-specific effect of ZBTB proteins in modulating gene expressions and cellular functions in T-cell compartment. Elucidating/identifying (i) how the expression of ZBTB TFs is transcriptionally/epigenetically regulated, (ii) how ZBTB proteins are post-translationally modified (i.e., phosphorylated & ubiquitinated, etc), (iii) what partners interact with ZBTB proteins, and (iv) how these modifications/interactions regulate the stability, transcriptional activity and specificity of ZBTB proteins, will shed new light on our understandings of their action mechanisms along the T-cell development and differentiation program. Notably, other functions, beyond chromatin remodeling and transcriptional activity, of ZBTB proteins should not be ignored as they may also directly participate in cellular processes like DNA-repair as reported for ZBTB24 (23). The combination of structural/computational studies, immunoprecipitation-mass spectrometry (IP-MS), epigenome (i.e., using the Assay for Transposase-Accessible Chromatin/ATAC technique) alongside transcriptome sequencing analyses will provide new insights into the assembly of ZBTB-containing protein complex and their mechanics of chromatin remodeling/transcription-dependent and -independent activities. Finally, inducible and cell/stage-specific manipulations of ZBTB expression/function would help clarify its significance under different *in vivo* conditions.

Notably, although ZBTB is a conserved family of TFs, their functions may differ substantially among different species. For instance, ZBTB24 is apparently dispensable for human embryonic development, while its deficiency is embryonic lethal in mice (176). Therefore, detailed longitudinal characterizations of immune cells in individuals with dysfunctional ZBTB16, ZBTB20 or ZBTB24 (47, 163, 177), in combination with *in vitro* knockdown/overexpression approaches and sequencing techniques, will provide invaluable insights into fundamental cellular pathways and functions regulated by ZBTB members in health and disease.

## Author Contributions

ZYC and TTH collected literatures and drafted the manuscript. ZYC and JW generated the figures. XMG discussed the literatures and revised the manuscript. YZ and JW designed the work, collected literatures, drafted, and revised the manuscript. All authors contributed to the article and approved the submitted version.

## Conflict of Interest

The authors declare that the research was conducted in the absence of any commercial or financial relationships that could be construed as a potential conflict of interest.

## Abbreviations

HSCs, hematopoietic stem cells; LMPPs, lymphoid-primed multipotent precursors; ELPs, early lymphoid precursors; CLPs, common lymphoid progenitors; TFs, transcriptional factors; DN, CD4^-^CD8^-^ double-negative; DP, CD4^+^CD8^+^ double-positive; SP, single-positive; DLL, delta-like; ETPs, early T cell progenitors; Treg, regulatory T; iTreg, induced regulatory T; iNKT, invariant nature killer T; MAIT, mucosal associated invariant T; MR1, MHC-I related protein 1; Tn, naive T; Tc, T cytotoxic; Te, effector T; Tm, memory T; ZF, zinc finger; ILCs, innate lymphoid cells; INTs, CD69^+^CD4^+^CD8^low^ intermediate thymocytes; BM, bone marrow; Tem, effector memory T; Tcm, central memory T; GC, germinal center; ICF2, Immunodeficiency, Centromeric instability, and Facial anomalies syndrome 2.
